# Gold Mining in Ecuador: A Cross-Sectional Assessment of Mercury in Urine and Medical Symptoms in Miners from Portovelo/Zaruma

**DOI:** 10.3390/ijerph14010034

**Published:** 2016-12-30

**Authors:** Paul Schutzmeier, Ursula Berger, Stephan Bose-O’Reilly

**Affiliations:** 1 Institute and Outpatient Clinic for Occupational, Social and Environmental Medicine, WHO Collaborating Centre for Occupational Health, University Hospital Munich, Munich 80336, Germany, Stephan.Boeseoreilly@med.uni-muenchen.de; 2 Department of Medical Information Sciences, Biometrics, and Epidemiology IBE, Ludwig-Maximilians-University Munich, Munich 81377, Germany; berger@ibe.med.uni-muenchen.de; 3 Institute of Public Health, Medical Decision Making and Health Technology Assessment, Department of Public Health, Health Services Research and Health Technology Assessment, UMIT (University for Health Sciences, Medical Informatics and Technology), Eduard Wallnoefer Center I, A-6060 Hall i.T., Innsbruck 6060, Austria

**Keywords:** mercury, artisanal small scale gold mining, Ecuador, urine, medical score sum

## Abstract

Mercury is a toxic metal and is used in small scale gold mining. In Portovelo, Ecuador, mercury has been an environmental and health problem for decades. The target of this study was to assess the mercury concentration in the urine of miners from Portovelo/Zaruma to establish a prevalence of high values. Eight hundred and sixty-five (865) urine samples were collected and analysed for their mercury content, using cold vapor atom absorption spectroscopy. The prevalence of high mercury values (>25 μg/L) was estimated. Forty-four (44) miners with mercury levels >15 μg/L filled in a questionnaire for characteristics and possible confounders, and were examined for intoxication symptoms to establish the ten points medical score sum. The median urine value was 1.8 μg/L; 78.3% of miners were below 7 μg/L and were not at risk of an intoxication, whereas 5.9% of miners exceeded the limit of 25 μg/L and were probable to experience intoxication symptoms. The medical score sum had a range of 2 to 8 points with a median of 6. The low prevalence of high mercury concentrations shows that the politics and techniques to eliminate the use of mercury are being successfully implemented. Further studies are needed to identify factors enabling this process.

## 1. Introduction

All around the world, gold is one of the most precious and most wanted metals, therefore, there is a high economic interest in producing it. Most of the production is done mechanically and on an industrial scale, however, there is also a significant portion that is carried out by manual labour. According to estimations, about 15% of gold (~400 tons) is mined manually by 10 to 15 million miners in 70 countries who work in artisanal and small scale gold mining (ASGM), including about 3 million women and children [1]. People in the ASGM sector work mostly informally, or even illegally, and usually belong to the poorer part of the population [2].

In ASGM, mercury is commonly used to extract the gold from the ore [3,4]. Once the ore is obtained from underground or open pit deposits, it needs to be crushed and ground into a fine powder. Usually, this is done mechanically by using simply constructed ball mills. The miners then process the powder by adding liquid mercury to extract the gold from the rock dust and dirt through amalgamation. This is a chemical reaction, in which only the gold particles bind to the mercury and form a solid compound. In the following step, this amalgam is smelted, usually with a blow torch by the, so called, amalgam smelters. Whilst the gold turns liquid in the heat, the mercury evaporates into the air, and a raw gold nugget remains after cooling. During the two processing steps the workers are exposed to the poisonous metal either by skin contact during the amalgamation or by inhalation during the smelting. Mercury is a toxic heavy metal and chronic exposure causes several health problems in members of mining communities [5,6,7,8,9,10].

The extensive use of mercury in ASGM accounts for 37% of the mercury emissions to air and water worldwide, and amounts to 727 tons per year [3]. This makes the ASGM sector the number one anthropogenic mercury pollutant in the world and demonstrates the risk it poses, not only to miners, but by distribution via water and air, also to the wider population and the environment. In terms of world regions, South America ranks third in emissions by ASGM, after Southeast/East Asia and Sub-Saharan Africa [3]. One of South America’s mining countries is Ecuador, in which ASGM has been practiced since the end of the 70s. Even up until today, large and medium scale mining only play a minor role there, which probably was in favour of the ASGM development and increased its political support [11,12]. Gold and ASGM make up a considerable part of the Ecuadorian economy, and the sector is still growing. According to conservative estimations, at least 90,000 persons are directly involved in this sector and 2.7% of the population depend on it directly and indirectly [12]. With its first legalisation in 1974, the government acknowledged its economic importance, and since then, it was not only implemented into the overall mining sector regulation, but also considered in a specific regulatory framework, which was last reformed in 2009. Finally, in 2013, Ecuador, alongside many other countries signed the treaty of the Minamata Convention and declared to take measures to reduce or even to eliminate the use of mercury in ASGM. The Convention requires the implementation of a national action plan, which includes strategies to inform miners and communities, to protect vulnerable populations from exposure, and the use of health facilities for data collection and promotion.

The area of Portovelo/Zaruma is the oldest, but still the most important, centre for ASGM in Ecuador. About 3000 tons of ore are processed there daily, and the area has a history of extensive use of mercury [13] The majority of gold is extracted in “Chilean mills”, big steel containers with two to three massive, motor-driven cement wheels to crush the ore, and the water carrying the gold dust is lea through sluice boxes for concentration [13]. Prior to the ban of mercury, at that point, the dust was further concentrated and amalgamated by manual panning. The amalgam was then smelted in open furnaces [13]. Several studies have proven a significant impact of mercury use in ASGM on the environment and the health of the people living there, such as children with elevated mercury levels and neurocognitive deficits or pollution of aquatic systems [7,11,12,14,15]. In 2010, the estimated amount of released mercury was 1.5 tons, of which 70% went into the air. The air concentrations in the town of Portovelo were highly elevated and above the critical threshold level of the Agency for Toxic Substances and Disease Registry that year [16]. Currently, Ecuador is subject to international political pressure from Peru, because the mining wastes and tailings of the ASGM areas enter the Puyango River, which carries the pollutant over the border. This affects the soil quality, the aquatic life, and the health of people in Peru [17]. The amount of environmental and health damages was estimated to be up to 35 billion U.S. dollars and evidences the need for changes in the sector, such as the closure of ASGM sites and the creation of a centralised waste disposal system [17]. For all those reasons, the Portovelo/Zaruma region is suitable as an indicator for the current situation of mining in Ecuador.

The main aim of this study was to assess the mercury (Hg) concentration in urine of miners from the Portovelo/Zaruma area in Ecuador, to establish a prevalence of critically high values, and to compare it with other ASGM regions. Additionally, the characteristics of a sub sample with elevated mercury levels were analysed in detail.

## 2. Materials and Methods

This is an observational, epidemiological study with a cross sectional design. The exposition and prevalence of mercury concentrations above 25 μg/L urine of the miners in Portovelo and Zaruma, Ecuador was analysed as an indication for chronic mercury intoxication. The data used in this study was assessed during a pre-screening and screening phase of a pharmaceutical study, however, it is not directly related to the possible product.

### 2.1. Subject Selection

The population of interest in this study were people who work in mines, with an age of 18 to 65 years, in area of Portovelo, Ecuador. To reach as many persons as possible within the three weeks (11 August 2015 to 29 August 2015) of urine analysis, we cooperated with a local gold mining association. The association Aproplasmin established contact to the mine operators, promoted the study, and scheduled visits for sample collection at the mining sites. In those visits, the miners were informed by the study doctors about the aims of the study and asked for a voluntary urine sample.

**Figure 1:**
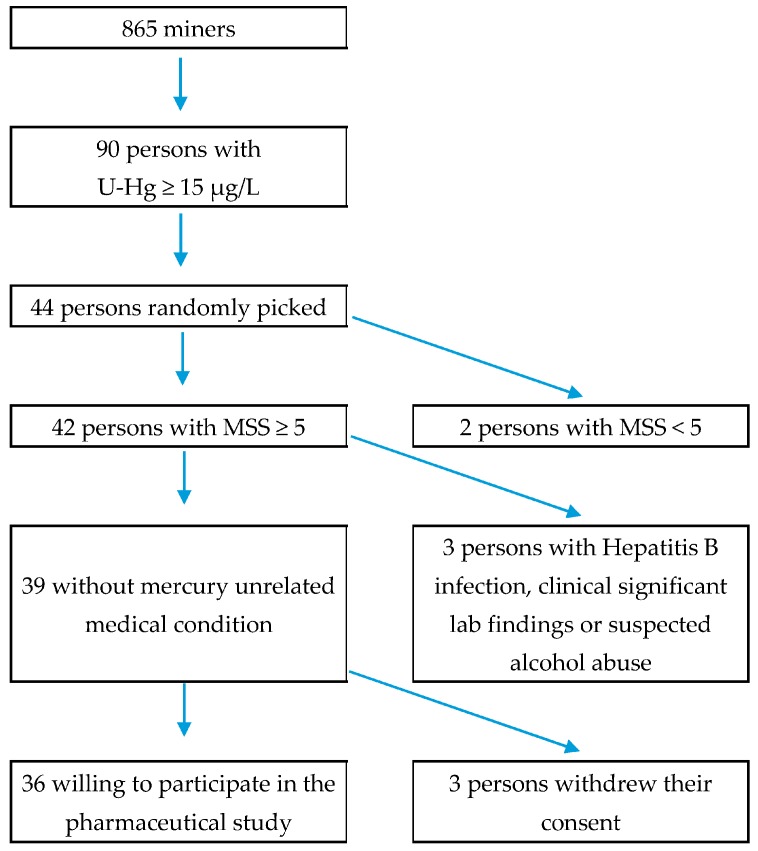
Flow chart of subject selection for the pharmaceutical study (MSS = chronic intoxication medical score sum).

For the conduct of the second part, persons with a urine Hg value above 15 μg/L were selected and examined further, if they qualified for the participation in the pharmaceutical study [18]. The exclusion criteria for the pharmaceutical study aimed at existing medical conditions in the participants: renal disorders or failures, abnormal results in haematology results, neurodegenerative disorder, drug or alcohol abuse, pregnancy, serious infections, allergies to the drug or its components, and participation in another drug study, which only were relevant for the experimental treatment. Persons that exceeded the threshold limit (15 μg/L urine) were invited to the local study site in the chronological order that their urine samples had been examined, until the aimed sample size for the pharmaceutical study of 36 participants was reached. For all invited patients, the mercury intoxication medical score sum (MSS) was assessed with a questionnaire and a medical test [18,19]. Apart from a high Hg in urine level, the subjects were to have a medical score sum of over 5 or, alternatively, of 3 out of 10 plus 2 out of 6 further symptoms of a chronic intoxication (e.g., physical fatigue or memory loss) [19]. Further data were obtained for 44 persons, which included 36 participants and eight screening failures (Figure 1). Those eight were excluded from the pharmaceutical study because of mercury unrelated medical conditions, not meeting the MSS inclusion criterion, or because they were not willing to participate further (see Appendix A for details on exclusion criteria).

All participants were volunteers and had signed a written consent form for the pharmaceutical study and this project. EmeraMed Ltd. (Stockholm, Sweden) and FOMAT Medical Research (Guayaquil, Ecuador), the clinical research organisation responsible for all procedures in Ecuador, had fulfilled the country specific demands and regulations to perform the health assessment. The relevant national, state, and local regulations were obeyed, the appropriate regional health authorities and the national ministries of health had given all necessary permission, including extensive legal, formal, and ethical considerations. The study was conducted in accordance with the Declaration of Helsinki, and the protocol was approved by the ethics committee of the Universidad de San Francisco de Quito, Diego de Robles y Via Interoceanica, Quito, Ecuador. Aprobación Protocolo 2015-048E, 26 May 2015.

### 2.2. Collection of the Urine Samples

The miners were asked to give a sample of spontaneous urine into a cup and their names were codified. The code was written on the cup, separated from the form, to ensure a blinded analysis. Those samples were brought to the laboratory within half a day’s time to be analysed as soon as possible, therefore no further processing was necessary. Until they arrived at the lab, they were kept in air-conditioned cars to prevent heat induced degradation.

### 2.3. The Lumex Analyser RA-951+

The collected samples were measured with a Lumex^®^ mobile mercury analyser (RA-915+) with a RP-91 liquid attachment (Ohio Lumex Co., Solon, OH, USA), which was basically comprised of a pump unit and an impinger system with a separator. With the so called cold vapour atom absorption spectrometry method (CV-AAS), aliquots of the samples were put into the glass separator, where they reacted with the tin-II-chloride (SnCl_2_). The inorganic Hg from the samples was reduced in a redox reaction to its monoatomic form, which has a relatively high vapour pressure at room temperature. The vaporised metal was then transported by the pump in a constant air flow into the analytical cell, where it absorbed a part of the monochromatic light, 253.7 nm, of the electrodeless discharge lamp. When a greater amount of Hg was released, more light was attenuated within the analytical cell. The detector of the Lumex^®^ translated the absorption into a signal, the data processor recorded it and calculated the concentration from the area under the curve and the aliquot volume. The limit of detection was a concentration of 0.5 μg·Hg/L [20].

### 2.4. Preparation of the Chemical Solutions

Firstly, to produce the SnCl_2_ solution, the hydrochloric acid (HCl) needed to be diluted with water from a 30% concentration (Suprapur^®^, Merck KGaA, Darmstadt, Germany) to a 3% concentration. We used clean mineral water, which did not pose a problem because the CV-AAS method is robust against solved minerals in water.

Secondly, the mercury standard solution with a concentration of 1000 mg/L (Certipur^®^, Merck KGaA, Darmstadt, Germany) needed to be reduced to a concentration of 100 μg/L. This was done in two steps, 50 μL standard were diluted with water to a volume of 5000 μL, and to 50 μL of that dilution were added another 4950 μL of water. The formerly mentioned volumes were taken with two different Eppendorf pipettes^®^ (Eppendorf AG, Hamburg, Germany) (Volumes: 500–5000 μL and 10–100 μL).

Lastly, 5 grams of SnCl_2_ were weighed into a beaker glass and solved in 100 mL of the 3% HCl (0.264 mol/L) to obtain the solution for the reduction.

### 2.5. Measurement

On every day of analysis, the solutions were freshly produced and the Lumex^®^ spectrometer was calibrated. Three different amounts of the diluted standard solution were used to obtain the values for the Hg concentrations of 0, 5, 10, and 20 μg/L and analysed for 60 s. The program calculated a calibration line from these values. A second standard solution was used to validate that line to reduce systematic error.

After the calibration step, the urine samples could be analysed. Of each sample an equivalent (e.g., 0.5 mL) was put into the impinge system, filled with 10 mL of the SnCl_2_ in 3% HCl solution (5 g/100 mL), and in the same instant the measurement recording was started. The impinge tube walls were rinsed shortly with hydrochloric acid during the process to ensure that all the mercury could react and be vaporised.

If the resulting peaks in the spectrum were too flat and the line did not hit the zero line within about 60 s, the tin reactant was replaced and the measurement was repeated for better accuracy. For very high concentrations, the analysis was redone with a smaller amount of the sample to get results within or closer to the calibration line and to improve the precision. After each day of measurements, the glass parts of the machine were extensively cleaned [20].

### 2.6. Threshold Values

The threshold values for mercury in urine are based on the recommendations of the Human Biomonitoring Commission [21]. It distinguishes between two Human Biomonitoring levels, HBM-I and HBM-II, to categorise the exposure level. Concentrations below the HBM-I limit are considered harmless and are not known to cause adverse health effects. Values between HBM-I and HBM-II are posed as a warning sign and the exposure should be reduced or eliminated as far as achievable, since adverse health effects cannot be excluded [22]. Values above the HBM-II threshold indicate an increased risk for negative health effects, therefore a reduction of the burden level is essential and a medical examination is advised [21,22]. The two HBM levels for Hg in urine are 7 μg/L and 25 μg/L. Additionally the threshold value of the biological exposure indices (35 μg/L), a recommendation of the American Conference of Industrial Hygienists, was examined.

For the recruitment of patients, initially, the threshold in the inclusion criteria had been set to 25 μg/L, accordingly to the HBM-II level, but was then lowered to 15 μg/L after the pre-screening to obtain sufficient participants. This threshold reduction is based on the Lumex^®^ analyser only measuring the inorganic mercury and thus tending to underestimate the total Hg level by about 25% [20]. This value in combination with a medical score sum of ≥3 has proven to identify intoxicated subjects in mining (Figure 1) [18].

### 2.7. Medical Score Sum and Further Clinical Symptoms

The test for the chronic intoxication medical score sum (MSS) is based on studies that identified significant symptoms for an intoxication [5,18]. Out of those symptoms, ten diagnostic items were selected with statistical methods to create an assessment tool for intoxication cases, which was utilized in our study [19]. Those items were established in three categories: anamnestic data (excessive salivation, tremor at work, sleeping problems), clinical symptoms (discoloration of gingiva, ataxia of gait, dysdiadochokinesia, heel-to-shin test, proteinuria) and neuro-psychological tests (Match box test, Pencil tapping test) [23]. Each of the previous items was binary coded, so that for each applying criteria or positive test result one score point was added. Accordingly, the highest achievable score sum was 10 (see Appendix B for more details).

Furthermore, six more symptoms for chronic intoxications were recorded for subjects with an Hg in urine level >15 μg/L after a protocol amendment, which were: metallic taste, irritability, social nervousness and/or withdrawal, memory loss, mental and physical fatigue. Memory loss was established with the Digit Span test after Wechsler [24] and the last two were assessed with a special score system [25]. Additionally, the mental and physical fatigue screening score was assessed. Patients could maximally achieve 16 points for physical and 10 points for mental fatigue [25]. Furthermore, the subjects were screened for alcohol by a breath test and drug abuse by urine analysis.

All previously mentioned diagnostics were done by local doctors, who were advised and trained on all the questions and tests.

### 2.8. Statistical Methods

All analyses were done with the statistical software R 3.0.0 (The R Foundation for Statistical Computing, Vienna, Austria). For statistical analysis, mercury in urine values below the limit of detection were set to half of it, which was 0.25 μg/L. The prevalence and the density of the mercury in the distribution of urine values were estimated with a Gaussian kernel.

Urine values, MSS, and population characteristics of the 44 persons were described stratified to exposure and jobs. Type of job, amalgam fillings, and symptoms were each described by frequencies and in percentages.

All characteristics were tested for normality by using graphical methods (e.g., histograms) and the Shapiro-Wilk method. The Kruskal-Wallis test was used to examine for differences in the variables across the exposed groups. To identify associations of the outcome variables and individual characteristics and type of job, the four job categories were dummy coded. Since some participants had more than one job, we tested each job group against all persons not belonging to that group employing the Mann-Whitney-U-test. Boxplots were used to present the distribution of mercury in urine for different groups.

The correlation between metric characteristics and the MSS and the Hg values were estimated using the robust Spearman’s correlation coefficient, since the distribution of the urine values is non-parametric and the MSS is an ordinal variable. The factors of interest were the number of years working in mining and the number of years living in the area. In addition, we performed a correlation analysis of weight, age, and the number of amalgam tooth fillings to identify confounders.

## 3. Results

### 3.1. Prevalence and Distribution

**Figure 2:**
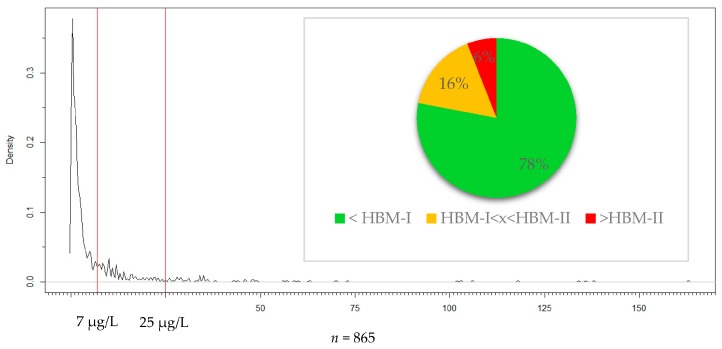
Estimated density distribution of the Ecuadorian mining population together with the HBM-I and HBM-II thresholds and proportions of persons in each Human Biomonitoring (HBM) category.

The number of persons tested for mercury was 865. The maximum value that was measured was 163 μg/L, while the minimum was below the limit of detection (0.5 μg/L) in 190 samples. The median value of the population was 1.8 μg/L and the 95th percentile was 28 μg/L (Table 1). Figure 2 shows the graph of the estimated density distribution. Testing results revealed that 78.3% (*n* = 677) of the miner population was below the HBM-I threshold (7 μg/L) and not at risk of an intoxication. Values between HBM-I and HBM-II were measured for 15.8% (*n* = 137) of the miners and 5.9% (*n* = 51) exceeded the HBM-II limit (25 μg/L) and accordingly were probable to experience intoxication symptoms (Figure 2). Results indicated that 3.4% of the miners had values above the biological exposure index of 35 μg/L. The distribution of the mercury in urine values was not normal, but showed tailing to the right side which means that while many of the miners had low values, few had very high mercury levels. For this reason, in addition to the arithmetic mean, the median was given.

### 3.2. Population Characteristics and Medical Score Sum

Ninety (90) of the eight hundred and sixty-five (865) workers (10.4%) had values above 15 μg/L, of which 44 were picked and examined further for screening purposes. This group had urine values ranging from 15 to 163 μg/L, while the mean was 50.8 μg/L and the median 35 μg/L. All of those were male. The age ranged from 19 to 59 years, with a mean of 38.6 years. The time workers have been living in the area ranged from 1 to 54 years, with a mean of 22.2 years, whilst the time they have been working in the mines ranged from 0.5 to 40 years, with a mean of 11.2 years (Table 2). As for possible confounders, 29 persons ate fish more than once a week and 7 miners had amalgam fillings (Table 3). There was no significant difference in the Hg values or MSS between the two groups of fish consumption and neither between the groups with and without tooth fillings. The distribution of weight was bimodal with a median of 81.6 kg. None of the subjects tested positive for alcohol.

**Figure 3:**
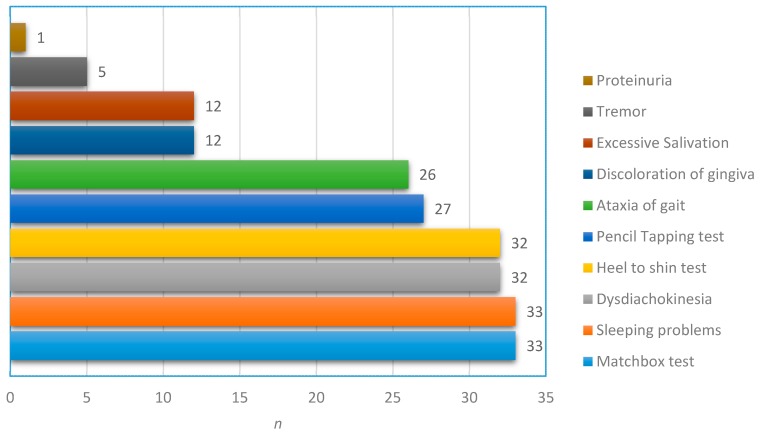
Frequencies of medical score symptoms and positive test results within subgroup (*n* = 36).

The chronic intoxication medical score sum had a range of 2 to 8 out of 10 points with a median of 6 and a mean of 5.8 points. The most common signs for an intoxication in the 36 patients were sleeping problems (91.7%), dysdiadochokinesia (88.9%), ataxia of gait (72.2%), and especially the neuro-psychological tests proved to be very sensitive: matchbox test (91.7%), heel-to-shin test (88.9%), and pencil tapping test (75%) (Figure 3). The data about the symptoms were only available for the 36 study patients. The assessment of further chronic symptoms was implemented after a protocol amendment and included 24 persons. Of those, physical and mental fatigue (each 62.5%) were the most frequent, followed by social nervousness and/or withdrawal and easy irritability (both 54.2%). The fatigue scores ranged from 0 to 11 out of 16, with a median of 3.5, for physical fatigue and from 0 to 5 out of ten, with a median of 1.5, for mental fatigue.

**Figure 4:**
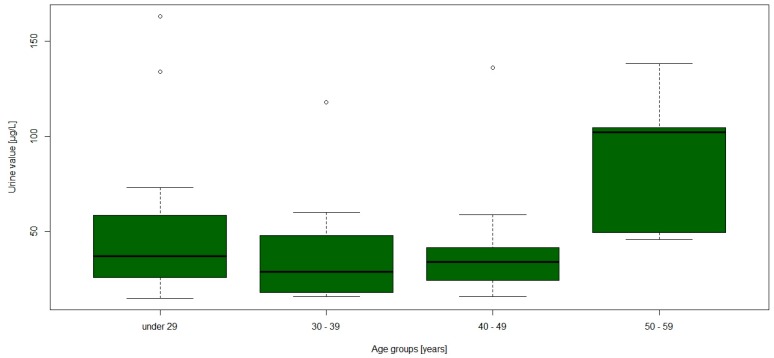
Boxplot of mercury in urine values stratified by age.

**Figure 5:**
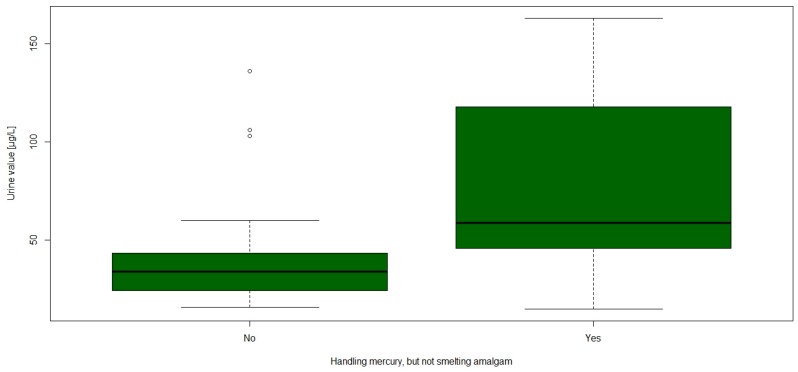
Boxplot of mercury in urine stratified by the job category “Handling mercury, but not smelting amalgam”.

The groups stratified by age showed a significant difference in the urine values, especially the oldest age group seemed to have higher urine values (Figure 4). Between the groups stratified by job, only one significant difference was apparent: the participants that were only handling mercury had significantly higher urine Hg values than the rest (*p* = 0.015, see Figure 5).

Spearman’s correlation analysis confirmed a significant weak to moderate correlation of the Hg values in urine and the MSS (*rs* = 0.381, *p* = 0.01). All the correlations were not statistically significant. Weight was weakly negatively correlated to Hg in urine (*rs* = −0.255, *p* = 0.094), whereas age showed a weakly positive correlation with MSS (*rs* = 0.259, *p* = 0.090).

## 4. Discussion

**Figure 6:**
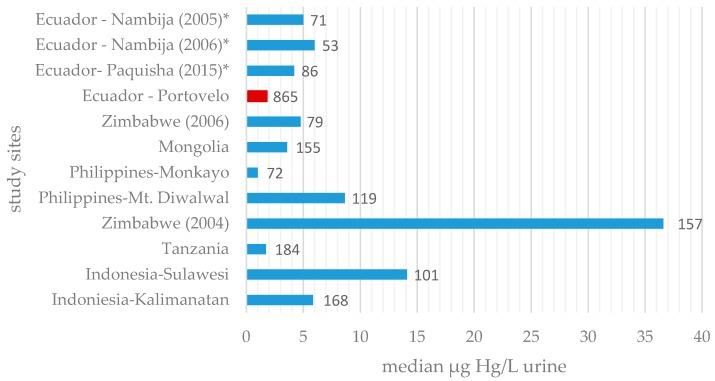
Mean mercury in urine values (μg/L) in comparison with other study populations [7,8,14,26,27]; The value of this study is marked red, numbers next to the bars are the number of participants. * Studies examined children in this area, not miners.

The estimated distribution showed strong tailing to the right side, as was shown in other countries as well, which means that most participants had low levels and only few participants showed high to very high levels of mercury in urine (see Figure 1) [5,6,20]. In comparison with other ASGM areas previously mentioned, urine concentrations were very low, actually lower than expected. In a study from 2011, miners from eight different mining areas in six different countries were analysed and the Hg in urine assessed [8]. Furthermore, compared to the results of that study, the in Ecuador assessed median and the 95th percentile are third to the lowest and the maximum is fourth to the lowest (Figure 6). The pooled values from all those areas are each higher than those we assessed [8]. The reported results from that study were consistent with and could be shown in another study of the same kind [6].

Moreover, the study gives the number of exposed participants that exceeded the HBM-II threshold for those eight regions combined (200 of 1035 persons, 19.3%), which is 3.3 times higher than the prevalence in Portovelo and Zaruma [8]. In another study, where 200 Ecuadorian gold miners of coastal areas were examined, Harari et al. found that about 5.1% had urine values above 35 μg/L, whereas we found that only 3.4% of the miners exceeded that value [28].

In a different recent study in Ecuador, which took place in 2013/14, 7- to 12-year old children living in Amazonian ASGM regions were examined for Hg exposure. González-Merizalde et al. found a median urine level of 4.2 μg/L and a maximum of 116.6 μg/L, which poses a considerably high concentration in a potentially less exposed population, since the children did not work with or smelt mercury themselves, and thus were not directly exposed [26]. Similar conclusions were drawn in two different studies with children, in which the mean Hg values in urine were 13.3 μg/L and 10.9 μg/L (vs. 6.4 μg/L) in the Nambija region in Ecuador [7,14]. Another study from 1997 found even higher values in a small sample [27]. There are more studies that examined mercury levels of mining populations throughout South America, however, those very often used hair samples or in fewer cases finger nail samples to assess it [29,30].

In regards to intoxication symptoms, it appears that the exposed Ecuadorian population is strongly affected by them. Compared to the highly exposed populations in a study, which utilized the same measuring instruments, all the frequencies of the intoxication symptoms are much higher [31]. The Ecuadorian subsample described here showed higher values than the previously mentioned group, however, signs like dysdiadochokinesia, ataxia of gait, or a failed heel-to-shin test for ataxia occurred more than twice as often. On one hand this underlines the sensitivity of the applied methods, on the other hand it indicates a higher exposure to mercury in previous years.

### 4.1. The Mercury Ban and the Half-Life of Mercury

The possible reasons for the unexpectedly low prevalence of high Hg values in urine are numerous. First and foremost is the physiological elimination since the passing of a new mercury ban in Ecuador in May 2015, about three months prior to measuring the samples. Similar laws exist in other countries as well, but the Ecuadorian government seems to enforce the regulations quite strictly. During our research, the workers often feared punishment by law, if they would cooperate with the doctors or thought we were sent by the government. On one hand, this means that the use of mercury was strictly and critically reduced in the official mining sites, on the other hand, miners, who smelt amalgam illegally, might have avoided this study in fear of consequences.

The elimination of Hg in urine approximately has a half-life between 50 and 60 days [28,32,33] and is possibly shortened with long term exposure, however, the experimental proof for this remains to be lacking [32]. This would mean, that between the passing of the ban and the laboratory measurement, roughly one and a half half-lives had passed and, accordingly, with a strict enforcement could be an explanation for reduced Hg levels.

Another reason is the underestimation of the mercury levels with the Lumex^®^ by up to 25%, as shown by Bauml et al. [20]. However, this should only explain a fraction of the low values.

### 4.2. Limitations

A possible limitation for the measuring of the urine samples is the loss of mercury from the urine into the walls of the plastic urine cup. However, the amount of metal that transgresses within the few hours of transportation is very small and should not affect the overall results. Another factor that could have been reducing the amount of metal in the samples was the relatively high outside temperature, which could catalyse a degradation of the Hg. Nevertheless, since we could establish a local laboratory, no long transportation or international shipping was necessary, and the samples were measured within the same day. This way, the degradation was kept to a minimum.

Also, the Lumex^®^ spectrometer, with the reduction agent SnCl_2_, is only able to measure the inorganic Hg in the urine, which makes up approximately 75% of the total Hg [20], therefore the total burden on the miners was underestimated. Additionally, for practicability reasons, there was no adjustment for creatinine to compensate the differences in urine concentrations. The adjustment itself is advised, but critically discussed [34,35]. Unfortunately, this also restricts the comparability to other studies.

With the data from the further examined sub group, correlation coefficients could be calculated, but the small patient number meant that it was only possible to get results with limited significance. Especially, data from a group of people with low Hg values or a control would have been very helpful to show differences in characteristics and symptoms. However, the results were aimed to assess the miners’ situation and generate further hypotheses to investigate how to improve it.

Additionally, problems occurred with the analysis of the questionnaire answers. The options in the multiple-choice question about the kind of job the workers were doing in mining within the previous year were concepted to be exclusive. However, fourteen persons picked more than one possibility, which could indicate either a high rate of recent changes in job, but is a systematic error by the local investigators. In fact, nine of the thirteen people who picked “handling mercury, but not smelting” also chose “smelting amalgam” as a category, which explains why the number of jobs in Table 3 does not add up to 44. Moreover, due to the above mentioned change in laws and the workers’ behaviour, this question could be biased by wrongful answers. This, and the lack of diversity in jobs, would explain why there was no correlation between smelting and the urine values, unlike in other similar studies [8,29]. Additionally, it was very surprising that persons in the category “handling, but not smelting” had a higher mean mercury in urine level than people smelting. This is in contradiction of prior findings and is probably caused by the high percentage of persons picking more than one category. The values of the answers concerning the years the workers lived and worked in the mining area could be prone to recall bias, especially the latter obviously shows a number preference bias.

## 5. Conclusions

The assessed prevalence of high mercury concentrations in the urine of miners was lower than expected and low compared to other countries. However, intoxication symptoms were very common in patients with higher values. In consideration of the few data available, the exposure to mercury within the miner population seemed to have a decreasing tendency within the last years. The greatest influence on this improvement probably derives from a very supporting legal and political situation. Further studies are needed to identify the most effective factors that lead to an enabling environment for eliminating mercury from mining and improving the safety and health of small scale gold miners. Furthermore, the situation in Ecuador could help as a model for other countries and communities to elaborate strategies to implement cleaner and safer mining practices, without the use of mercury.

## Figures and Tables

**Table 1:** Summary of urine values (μg/L).

Parameter	Value (µg/L)
*n*	865
Minimum	<0.5 *
1st Quartile	0.7
Median	1.8
3rd Quartile	5.4
95th Quantile	28.0
Maximum	163.0

* Below the limit of detection.

**Table 2:** Summary of mercury related characteristics (*n* = 44).

Parameter	Urine-Hg (μg/L)	MSS	Years Working *	Years Living *
Minimum	15.0	2.0	0.5	1.0
1st Quartile	26.5	5.0	3.8	7.8
Median	35.0	6.0	9.0	22.0
Mean	50.8	5.9	11.2	22.2
3rd Quartile	57.5	7.0	16.0	32.0
Maximum	163.0	8.0	40.0	54.0

* In the mining area of Portovelo/Zaruma; MSS = chronic intoxication medical score sum.

**Table 3:** Stratification of characteristics by job category.

Parameter	Another Job	Gold Buyer	Handling Only	Smelting
*n*	3	4	13	37
Urine				
Minimum	16.0	16.0	15.0	15.0
1st Quartile	23.0	25.8	46.0	27.0
Median	30.0	30.0	59.0	35.0
3rd Quartile	51.5	57.3	118.0	50.0
Maximum	73.0	136.0	163.0	163.0
W *	73.5	93.5	106.5	149
*p*-value	0.592	0.595	0.015	0.542
MSS				
Minimum	5.0	4	5	2
Median	5	5.5	6	6
Maximum	5	7	7	8
W *	93	93.5	164	95
*p*-value	0.136	0.584	0.326	0.260
Years working in the mining area				
Minimum	3	0.5	1	0.5
Median	5	5.8	10	10
Maximum	14	16	20	40
*W* *	73.5	106	206.5	80
*p*-value	0.591	0.297	0.908	0.115
Years living in the mining area				
Minimum	6	1.5	3	1
Median	29	26	25	22
Maximum	32	42	54	54
W *	55	75	193	116
*p*-value	0.780	0.854	0.837	0.676
Age				
Minimum	29	32	25	19
Mean	32	39.8	39.9	39.3
Maximum	35	46	56	59
W *	86.5	73.5	179.5	82.5
*p*-value	0.253	0.806	0.580	0.135
Fish consumption				
>1/week	2 (66.7%)	3 (75.0%)	7 (53.8%)	25 (67.6%)
Weight				
Minimum	70	63	54.5	63
Median	87.5	74	78	77
Maximum	96	82	107	96
W *	60	111	258	95.5
*p*-value	0.963	0.213	0.150	0.282
Amalgam fillings				
persons with AF	0	0	0	7 (18.9%)

* Wilcoxon rank sum statistic; MSS: chronic intoxication medical score sum.

## Appendix A. List of Exclusion Criteria

Subjects could not be entered in the study if any of the following exclusion criteria were fulfilled:

1. History of any clinically significant disease or disorder which, in the opinion of the investigator, may either put the subjects at risk because of participation in the study, or influence the results or the subject’s ability to participate in the study.
2. Known or a medical history of renal disorder, significant renal failure, or high risk of renal failure.
3. Any clinically significant abnormalities in clinical chemistry or haematology results at the time of screening as judged by the investigator.
4. Known or suspected neurodegenerative disorder including but not limited to stroke, polio, Parkinson’s and Alzheimer’s disease.
5. Known or suspected drug or alcohol abuse.
6. Positive pregnancy test in women.
7. Serious bacterial and chronic viral infection such as human immunodeficiency virus (HIV) or hepatitis virus.
8. History of severe allergy/hypersensitivity or on-going allergy/hypersensitivity, as judged by the investigator or history of hypersensitivity to drugs with a similar chemical structure or class to NBMI.
9. History of allergy/hypersensitivity to bisulphites (e.g., red/white wine).
10. Participation in any other clinical study that included drug treatment within three months of the first administration of investigational product.
11. Use of other therapies for mercury intoxication including metal chelators within three months.
12. Investigator considers subject unlikely to comply with study procedures, restrictions, and requirements.

## Appendix B. Medical Score Sum Assessment Tool

**Parameter**	**Score**
Anamnestic Data	
1. Excessive salivation	○ 0 (Not more than once a week) ○ 1 (At least once a week) ○ 1 (At least once a day)
2. Tremor at work	○ 0 (No tremor/tremor does not interfere with job) ○ 1 (Able to work but need to be more careful than an average person) ○ 1 (Able to do everything but with errors, poorer than usual performance because of tremor or more)
3. Sleeping problems at night	○ 0 (Feels ok after usual night of sleep) ○ 1 (Feels medium after usual night of sleep) ○ 1 (Feels bad after usual night of sleep)
Clinical Data	
4. Bluish discoloration of gingiva	○ 0 (No) ○ 1 (Yes)
5. Ataxia of gait	○ 0 (Absent) ○ 1 (Slight, ataxia only visible when walking on tandem or without visual feedback) ○ 1 (Moderate, ataxia is visible in normal walking, difficulties when walking on tandem)
6. Dysdiadochokinesia	○ 0 (Absent) ○ 1 (Slight, minimal slowness of alternating movements) ○ 1 (Moderate, marked slowness of alternating movements or more)
7. Heel-to-shin test	○ 0 (Absent) ○ 1 (Slight hypermetria in heel-to-shin test) ○ 1 (Moderate, hypermetria and slight ataxic performance of heel-to-chin test or more)
8. Proteinuria	○ 0 (Negative or traces) ○ 1 (Positive)
Neuro-psychological tests	
9. Match box test |__|__|__| s	○ 0 (≤ 18 s) ○ 1 (> 18 s)
10. Pencil tapping test |__|__| taps	○ 0 (>45 taps) ○ 1 (≤45 taps)
Medical Score Sum |__|__|	
